# Bimatoprost in the Treatment of Eyelash Universalis Alopecia Areata

**DOI:** 10.4103/0974-7753.77511

**Published:** 2010

**Authors:** Teresa Ojeda Vila, Francisco M Camacho Martinez

**Affiliations:** Universital Hospital Virgen Macarena, Sevilla, Spain

**Keywords:** Alopecia areata, bimatoprost, eyelash growth, prostaglandins

## Abstract

**Objectives:**

To evaluate topical bimatoprost for eyelash growth in patients with alopecia areata (AA).

**Design:**

A 1-year retrospective study, bilateral eyelash alopecia.

**Materials and Methods:**

Forty-one subjects with AA universalis without ocular disease applied 0.03% bimatoprost to the eyelid margin once a day over the course of 1 year.

**Results:**

Thirty-seven subjects completed the study, one patient was eliminated due to conjunctivitis at the beginning of treatment, two patients developed conjunctivitis after 6 months of treatment, and a fourth did not follow directions. Researchers evaluated patients’ eyelash growth every 4 months. We observed complete growth in 24.32%, moderate growth in 18.91%, slight growth in 27.02% and without response in 29.72%.

**Conclusion:**

Bimatoprost may be effective and safe in the treatment of eyelash AA. 43.24% of the patients had an acceptable cosmetic response (total and moderate growth).

**Limitations:**

Design without control.

## INTRODUCTION

Eyelash and eyebrow hypotrichosis is a common sign of alopecia areata (AA) totalis or universalis. This may cause decreased protection to the eyes and be cosmetically detrimental for patients. Prostaglandin analogs are the treatment for the glaucoma and elevated intraocular pressure (IOP). This treatment was incidentally found to treat hypotrichosis eyelashes. In 1997 Johnstone[1] reported the first case of hypertrichosis of the eyelashes by topical latanoprost and since then there have been several reports about the prostaglandin analogs as treatment to eyelashes in AA. In December 2008 the US Food and Drug Administration approved 0.03% bimatoprost solution (Latisse^®^), identical to the ophthalmic solution for glaucoma treatment (Lumigan^®^), for increasing eyelash length, thickness and darkness in patients with hypotrichosis of the eyelashes,[2] or persons who desire lengthy eyelashes.

## MATERIALS AND METHODS

We performed a 1-year retrospective study, and non-blinded with patients who had AA universalis. The study was developed in the Trichology Unit of Department of Dermatology of our Hospital where we treat the affected scalp and eyebrows with triamcinolone acetonide in saline solution at 0.125 every 3 months. The wash-out period for other treatments for the eyelashes AA was of four months. All subjects underwent an ophthalmic examination at the beginning and at the end of the study period. Patients applied topical 0.03% bimatoprost ophthalmic solution once a day at night on the eyelid margins bilaterally with a cotton applicator or their finger. They were seen for a follow-up examination every 4 months. In each review, including the basal visit, standardized digital photographs of the eyelashes were taken with a DermLite photo adapted to a Nikon Coolpix 4500, and every patient was evaluated by one investigator (T.O.) based on the picture taken in each visit.

A total of 41 patients were enrolled for the participation at the beginning, but we completed the study with 37 patients completed the study. Out of four patients excluded from the study one had conjunctivitis in the first month of treatment and two had after 6 months, none developed glaucoma.

The level of response was grouped in four categories: no regrowth, slight regrowth (1-29%), moderate (30-80%) and total (>80%). This classification was used and accepts by others authors in various studies.[3,4] Investigators and subjects considered a cosmetic response to the moderate and total regrowth.

## RESULTS

**Figure 1:**
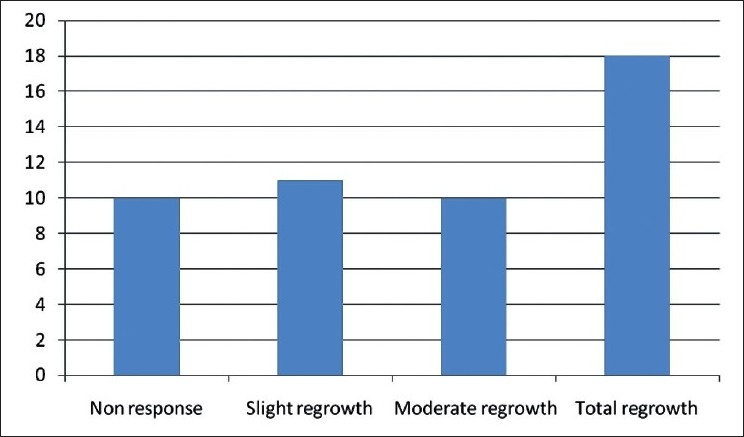
Responses to bimatoprost 0.03%

**Figure 2:**
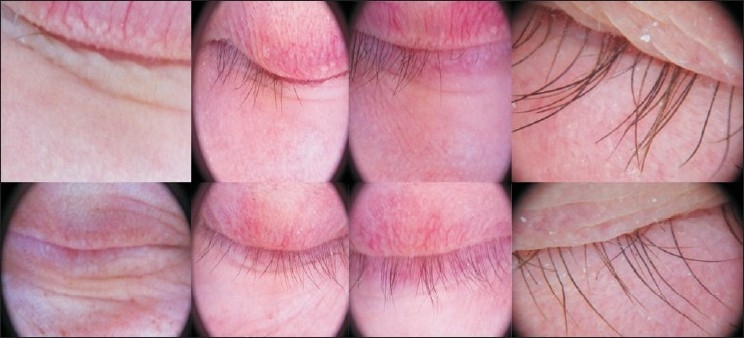
Moderate regrowth after 1 year of using bimatoprost

**Figure 3:**
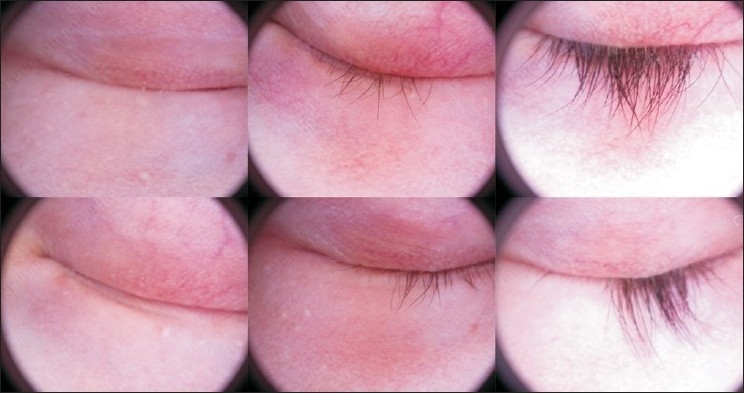
Moderate regrowth after 8 months of treatment with bimatoprost

**Figure 4:**
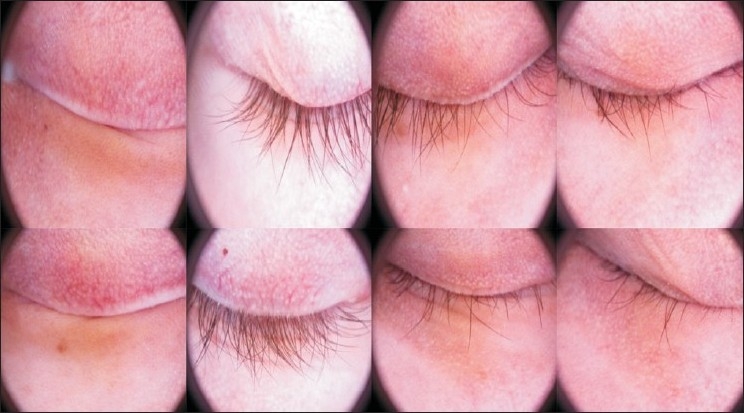
Recurrence during the treatment

There were 16 women and 25 men. Five patients were children less than 14 years, 38 had dark eyes and only three had pale eyes. At the end of the study (1-year follow up) 24.32% of subjects showed a complete regrowth, moderate growth was reported in 18.91%, slight growth in 27.02% whereas no response in remaining 29.72% [Figure 1]. Sixteen subjects (43.24%) got a cosmetically acceptable response [Figure 2] evaluated by the investigators and to the patients’ satisfaction, and thirteen of these subjects got there growth of eyebrows. The average time needed to induce eyelash regrowth was from 4 to 8 months. Twenty-six subjects achieved a moderate or total regrowth [Figure 3] when seen for their 8-month check up. During the study, recurrences occurred in four patients (10.25%), all with a slight final response [Figure 4]

Concerning side effects, three patients could not finish the study due to conjunctivitis, and all of them were then treated with latanoprost. We did not find any hyperpigmentation in the eyelid or iris, nor any cases of eyelid pruritus or redness.

### Comments

AA is an autoimmune disease affecting any hair-bearing area of the body, including the eyelashes. Currently no therapies are specifically targeted for the regrowth of eyelashes in these patients.

Prostaglandin analogs (latanoprost, bimatoprost and tavoprost) have been well-established as an IOP lowering agents for glaucoma since 1996.[5] Bimatoprost has a chemical structure similar to PGF2a analogs, although it is not a prostaglandin, and the free acid of bimatoprost is very similar to that of latanoprost.[6] Among side effects on the eyelashes are the following: increased length, thickness and pigmentation.[7] The exact mechanism of action of prostaglandins analogs in the hair follicle is not very clear yet. It is supposed that these drugs develop eyelash hypertrichosis because they are targeted at the hair follicle prostaglandin receptor[8] and may also prolong the anagen phase of eyelashes, leading to an increase of its length.[9] Many ophthalmologists observed on several occasions the growth of eyelashes in “healthy patients” Wester *et al*.[10] reported a randomized controlled trial with 19 subjects. Each participant received two vials of gel suspension, which contained bimatoprost and normal saline, respectively, and labeled “right eye” and “left eye” according to randomization. The suspension was applied to the upper eyelashes every evening on the designated eye for 6 weeks. The mean eyelash regrowth from baseline in the bimatoprost group was 2.0 mm versus a mean of 1.1 mm in the placebo group, which was a statistically significant difference (*P*=0.009). But the importance in Dermatology is the effects of these prostaglandins analogs (latanoprost, bimatoprost and tavoprost) in eyelashes hypotrichosis as is habitual in AA [Table 1]. Coronel-Pérez *et al*.[4] reported a 2-year prospective study of 54 patients with AA universalis. A control group comprised of 10 subjects who received corticosteroids treatment in eyebrows and scalp, and a treatment group included 44 subjects who received the same treatment as control group and latanoprost ophthalmic solution on the eyelid margins every night. they obtained complete regrowth in 17.5% and moderate regrowth in 27.5%, so it is concluded latanoprost could be an effective drug for eyelash AA. There were no side effects.

**Table 1:** Summarizes all the literature on prostaglandin agonist treatment in alopecia areata

Authors	Numbers of patients	Follow-up	Results
Ochoa *et al. JAAD* 2009	7	16weeks	Regrowth in two patients
Roseborough *et al. JAAD* 2009	11	16 weeks	No changes
Coronel-Pérez *et al. JEAD* 2010	44	2 years	Complete regrowth in 17.5%; Moderate regrowth in 27.5%
Zaheri *et al. Clin Exp Dermatol* 2010	1	8 weeks	Pronunced lash growth

Ochoa *et al*.,[5] after Roseborough *et al*. reporting on a 16-week controlled study with bimatoprost and latanoprost without changes in the growth of eyelashes of the eleven patients,[11] presented a 16-week prospective study of seven patients with AA, with an application of bimatoprost ophthalmic solution once daily. The solution was well-tolerated but not effective in promoting eyelash growth in five patients with 95% or greater eyelash loss caused by AA. Authors suggest that patients with less extensive eyelash loss caused by AA may benefit from treatment with bimatoprost applied, as seen in two patients.

Zaheri and Hughes[12] reported a 16-year-old girl with AA in eyebrows and eyelashes. She applied bimatoprost cutaneouslyonce a day to the eyelids and pronounced lash growth was noted at 8 weeks.

## CONCLUSIONS

We observed an acceptable response in 43.24% of patients with bilateral eyelashes AA using 0.03% bimatoprost ophthalmic solution, but we observed three patients with conjunctivitis. The limitations in this study are: we did not compare the treatment with other treatment or placebo, the subjects did not have the same basal situation, this is not a blinded study.
